# Is cardiac Troponin I Considered as A Predictor of In-hospital Mortality among COVID-19 Patients? A Retrospective Cohort Study

**DOI:** 10.30476/BEAT.2022.92719.1310

**Published:** 2022-01

**Authors:** Mohammad Haji Aghajani, Roxana Sadeghi, Reza Miri, Mohammad Parsa Mahjoob, Fatemeh Omidi, Fatemeh Nasiri-Afrapoli, Asma Pourhosseingoli, Niloufar Taherpour, Amirmohammad Toloui, Mohammad Sistanizad

**Affiliations:** 1 *Prevention of Cardiovascular Disease Research Center, Shahid Beheshti University of Medical Sciences, Tehran, Iran*; 2 *Physiology Research Center, Iran University of Medical Sciences, School of Medicine, Tehran, Iran*; 3 *Department of Clinical Pharmacy, School of Pharmacy, Shahid Beheshti University of Medical Sciences, Tehran, Iran*

**Keywords:** Troponin I, Cardiac biomarker, COVID-19, Hospital mortality, Heart injuries

## Abstract

**Objective:**

To describe the levels of troponin I in COVID-19 patients and its role in the prediction of their in-hospital mortality as a cardiac biomarker.

**Methods:**

The current retrospective cohort study was performed on the clinical records of 649 COVID-19-related hospitalized cases with at leat one positive polymerase chain reaction (PCR) test in Tehran, Iran from February 2020 to early June 2020. The on admission troponin I level divided into two groups of ≤0.03ng/mL (normal) and >0.03ng/mL (abnormal). The adjusted COX-regression model was used to determine the relationship between the studied variables and patient’s in-hospital mortality.

**Results:**

In this study, the median age of subjects was 65 years (54.8% men) and 29.53% of them had abnormal troponin I levels. Besides, the in-hospital mortality rate among patients with abnormal troponin I levels was found to be 51.56%; whereas, patients with normal levels exhibited 18.82% mortality. Also, the multivariable analysis indicated that the risk of death among hospitalized COVID-19 patients displaying abnormal troponin I levels was 67% higher than those with normal troponin I levels (Hazard ratio=1.67, 95% confidence interval=1.08-2.56, *p*=0.019).

**Conclusion:**

It seems that troponin I is one of the important factors related to in-hospital mortality of COVID-19 patients. Next, due to the high prevalence of cardiac complications in these patients, it is highly suggested to monitor and control cardiac biomarkers along with other clinical factors upon the patient’s arrival at the hospital.

## Introduction

Corona Virus Disease 2019 (COVID-19) is a serious and deadly infectious disease caused by severe acute respiratory syndrome coronavirus 2 (SARS-CoV-2) [1]. Moreover, with the disease’s outbreak of late 2019, the COVID-19 pandemic spread around the globe. The magnitude of the disease was more than 35 million cases such that by the end of September 2020, and beyond one million deaths had been recorded worldwide. Moreover, October 2020 marks the 2^nd^ wave and in some countries the 3^rd^ wave of the disease [2]. Also, COVID-19 infection is sometimes accompanied by mild symptoms. However, certain symptomatic patients might exhibit conspicuous symptoms such as fever, cough, and fatigue during times. Also, certain patients may suffer serious life-threatening complications [3]. Approximately, 15% of COVID-19 patients experience severe pneumonia and other acute pulmonary complications arising from activation of inflammatory responses as well as oxidative stress. The aforementioned processes could result in acute respiratory distress syndrome, multiple organ failure and eventually death [4]. Furthermore, decreased levels of arterial oxygen saturation revealed in lung CT scan [5] that were major manifestations of COVID-19 in patients displaying the critical form of the disease. Also, mortality-related cases cover a wide array of hospitalizations ranging from 3.4% extending to even more than 50% in certain reports [6]. Next, this disease is a multi-organ disease which causes several complications by attacking different tissues [7-9].

A relatively common complication in COVID-19 patients especially in the elderly and patients with underlying predisposing factors such as hypertension or diabetes mellitus is cardiovascular lesions [10, 11], which manifest in the form of ischemic heart attacks, myocarditis, dysrhythmias, vascular and arterial disorders, and consequently death [12]. In this regard, a rise in the level of cardiac biomarkers such as troponin as a marker of myocardial damage has been fully proven. Moreover, it seems that COVID-19 associated symptoms of myocardial lesions and cardiac dysrhythmias can lead to a surge in the level of this biomarker, even in patients without prior history of cardiovascular disease. Thus, increased troponin levels in the above-mentioned patients may be a prognostic factor to predict the disease severity and mortality rate [13, 14]. Although some studies hint at a connection between troponin I levels and mortality in COVID-19 patients [15-17], their numbers are limited and the foregoing correlation is not well understood. Therefore, in this study, we are going to introduce and then investigate the hypothesis that increasing in troponin I levels could be a prognostic tool to predict mortality among hospitalized COVID-19 patients.

## Materials and Methods

*Study Design and Subjects*

The present study is a retrospective cohort study conducted from February 18 to July 20, 2020. Moreover, its data was collected from the clinical records of 991 COVID-19 patients admitted to Imam Hossein Hospital located in Tehran, Iran. Hospitalization criteria was based on detection of physician and national clinical pathway of COVID-19 patients. These criteria were based on clinical signs such as dyspnea or increase respiratory rate (≥30 breaths per min) or oxygen saturation ≤ 93% or decrease in saturation to <90% with ambulation. Furthermore, all the aforementioned patients’ PCR tests were positive for COVID-19. It is also important to note that patients with the clinical diagnosis of COVID-19, whose diagnosis was not confirmed by a PCR test were excluded from the study. Since certain patients were excluded from the study as a result of the required information lack, only the data from 649 patients with confirmed COVID-19 were evaluated. Also, the present study was approved by the ethics committee of Shahid Beheshti University of Medical Sciences.

*Data Collection*

At first, a researcher-made checklist was used to collect data from the patients’ clinical records. The checklist contained information such as demographic characteristics, laboratory results, clinical information, symptoms list upon hospital arrival, underlying diseases, patient ECG information, and the disease outcome such as in-hospital death and ICU admission. Moreover, an average obtained from three various measurement periods (hospital admission, mid-hospital stays and before discharge or death) was used in this study. The reported measurements included respiratory rate, pulse rate, body temperature, and oxygen saturation (SpO2) of patients in the 3 mentioned stages. On the other hand, the first laboratory report data within 24 hours of patient’s admission was used in this study even though some lab measurements might have been repeated during that the initial 24-hours period. Furthermore, the amount of troponin I was quantitatively analyzed by the lab unit of Imam Hossein Hospital using chemiluminescence technique and ORTHO Clinical Diagnostic Vitros Device Kits made in the USA, and the results were qualitatively divided into two groups: normal (≤0.03 ng / mL) and abnormal (>0.03 ng / mL).

*Outcome*

In this study, the primary outcome was in-hospital mortality and the patients were followed from admission to discharge or when the patient died during hospitalization. 

*Data Analysis*

After collecting the data, its accuracy was checked and the collected sample was assessed for duplicate and missing data. Furthermore, Qualitative data were expressed using frequency and percentage, and quantitative data were reported as the median and interquartile range (IQR). Also, the data normality was determined by utilizing Kolmogorov-Smirnov test (K-S test) and histogram. The difference between variables was specified via the employment of appropriate statistical tests such as student’s t-test or Mann Whitney U test and chi-square test. In addition, in order to investigate COVID-19 death-related factors and to control possible confounding variables, COX regression model was used at the univariate and multivariable levels. Moreover, to select the best variables for entering the cox model, a stepwise selection model was applied, which included a backward approach with a *p *value of less than 0.2. Furthermore, the investigation of cox analysis assumption (proportional hazards assumption) was done by applying Schoenfeld residual test. The Schoenfeld residual test applies the assumption that the risks are proportional to variables that have a *p*-value greater than 0.05. In addition, all statistical analysis were performed at a significance level of less than 0.05 using STATA 14 software and reported with 95% confidence interval.

## Results

Out of the 649 hospitalized cases whose COVID-19 was confirmed, 351 (54.08%) were men. Moreover, the median age of the subjects was 65 years (interquartile range: 52-76) and the median time of COVID-19-related hospitalization was 6 days (interquartile range: 4-10). Eighty-four patients (12.94%) were admitted into the intensive care unit (ICU) of the hospital. Of all the hospitalized patients studied, 185 (28.51%) expired in the hospital. Also, the most common symptoms among COVID-19 patients were gastrointestinal symptoms such as diarrhea, nausea, lack of appetite (anorexia), and abdominal pain (416 persons, 64.1%), shortness of breath (401 patients, 61.8%), and cough (320 patients, 49.3%), respectively. Furthermore, based on the initial ECG evaluation, 367 (56.55%) of the patients suffered from dysrhythmias at the time of admission to the hospital. In this study, hypertension (45.30%) had the highest prevalence among underlying diseases, followed by cardiac disease (42.22%) and diabetes (32.36%) as illustrated in Table 1.

**Table 1:** Comparing the baseline characteristics of hospitalized COVID-19 patients between Troponin I groups

**Variables**	**All patients** **(n=649)**	**Troponin I≤0.03, ng/mL (n=457)**	**Troponin I>0.03,** **ng/mL (n=192)**	**P-value**
Age (yrs)	65 (52-76)	60 (48–72)	74 (63–83 )	<0.001
Sex (Men)	351 (54.08)	238 (52.08)	113 (58.85)	0.114
Body Mass Index (BMI, Kg/m^2 ^)	25.96 (23.98–29.3)	26.21 (24.22–29.41)	25.39 (23.43–28.40)	0.038
Symptoms on admission (Yes)				
Chest pain	69 (10.63)	36 (7.88)	33 (17.19)	<0.001
Dyspnea	401 (61.79)	283 (61.93)	118 (61.46)	0.911
Myalgia	191 (29.43)	160 (35.01)	31 (16.15)	<0.001
Cough	320 (49.31)	244 (53.39)	76 (39.58)	0.001
Fatigue	245 (37.75)	177 (38.73)	68 (35.42)	0.427
Fever	315 (48.54)	239 (52.30)	76 (39.58)	0.003
Gastrointestinal Symptoms	416 (64.10)	311 (68.05)	105 (54.69)	0.001
Dysrhythmia	367 (56.55)	242 (52.95)	125 (65.10)	0.004
Duration of hospitalization (days)	6 (4–10)	7 (4–10)	6 (3–10)	0.047
Duration of hospitalization among alive patients (days)	7 (4–10)	7 (4–10)	6 (4–12)	0.527
Duration of hospitalization among dead patients (days)	6 (3–11)	7.5 (4–13)	4 (2–10)	0.001
ICU^b^ admission (Yes)	84 (12.94)	56 (12.25)	28 (14.58)	0.420
Ventilator (Yes)	103 (15.87)	52 (11.38)	51 (26.56)	<0.001
In-hospital Mortality (Yes)	185 (28.51)	86 (18.82)	99 (51.56)	<0.001
Medical assessment during hospitalization				
Pulse Rate (PR, pulse / min)	84.50 (79.33–92)	84.67 (79.67–91)	84 (79.33–95.67)	0.569
Respiratory Rate (RR, per 1/min)	19 (17.67–22)	19 (17.67–22)	19.33 (17.67–22)	0.697
SPO2 ^a^ (%)	91 (87.33–93.33)	91.33 (83.33–93.33)	90 (84–93)	0.004
Mean Arterial Pressure (MAP, mmHg)				
<70	19 (3.31)	5 (1.25)	14 (8.09)	<0.001
70-100	528 (91.99)	380 (94.76)	148 (85.55)	
>100	27 (4.70)	16 (3.99)	11 (6.36)	
Body temperature (°c)	37.06 (36.83–37.33)	37.07 (36.83–37.33)	37.07 (36.83–37.33)	0.807
Underlying diseases (Yes)				
Hypertension	294 (45.30)	171 (37.42)	123 (64.06)	<0.001
Cardiac diseases	274 (42.22)	147 (32.17)	127 (66.15)	<0.001
Diabetes	210 (32.36)	132 (28.88)	78 (40.63)	0.004
Chronic Kidney Diseases (CKDs)	79 (12.17)	34 (7.44)	45 (23.44)	<0.001
Cancer	28 (4.31)	23 (5.03)	5 (2.60)	0.165
COPD^c^	59 (9.09)	31 (6.78)	28 (14.58)	0.002
Laboratory values (In admission)				
Hemoglobin (g/dL)	12.5 (11.10–13.70)	12.6 (11.35–13.80)	12.2 (10.80–13.50)	0.026
White Blood Cell (10^9^/L)	6.9 (5.1–10.1)	6.5 (4.8–8.65)	9.4 (6.6–11.8)	<0.001
C-reactive protein (mg/L)	48 (21.9–75.4)	46 (21.9–72)	53.55 (22–90)	0.052
Procalcitonin (ng/L)	0.58 (0.3–1.53)	0.46 (0.27–0.93)	1.09 (0.41–3.15)	<0.001
Urea (mg/dL)	43.5 (31.8–63)	39 (28.3–53.7)	62.55 (43.15–96.75)	<0.001
Creatinine (mg/dL)	1.2 (1–1.7)	1.2 (1–1.4)	1.7 (1.2–2.3)	<0.001
Creatine Phosphokinase (CPK) (U/L)	117 (62–265)	95 (58–210)	163 (91–434)	<0.001
Creatine kinase-MB (CK-MB, U/L)	10 (1.3–23)	10 (0.9–21)	10.6 (2.6–30)	<0.001

^a^Oxygen saturation measured by pulse oximetry; ^b^ICU: Intensive Care Unit; ^c^COPD: Chronic Obstructive Pulmonary Disease; Values are n (%), median (Q1-Q3)

In general, the initial evaluation of patients revealed that 192 patients (29.53%) had abnormal troponin I (0.03 ng / mL <) with an in-hospital mortality of 51.56%; whereas, the in-hospital mortality rate for the group with normal troponin I levels was found to be 18.8%. 

Table 1 compares the baseline characteristics of studied patients between normal and abnormal troponin I levels. Median age and median laboratory factors such as procalcitonin levels (*p*<0.001), serum urea levels (*p*<0.001), creatinine (*p*<0.001), white blood cell count (*p*<0.001), median of other cardiac enzymes (CPK and CK-MB) (*p*<0.001) were significantly higher in subjects with abnormal troponin I level. Morever, median hemogolobin value (*p*=0.026) and median of oxygen saturation(SpO2) (*p*=0.004) in COVID-19 patients with abnormal troponin I were less than COVID-19 patients with normal troponin I level.

Also, distribution of variables such as in-hospital mortality, mean arterial pressure (<70 mmHg) and history of chronic kidney diseases (CKD) among COVID-19 patients with abonormal troponin I level were higher (*p*<0.001) (Table 1).

According to the results of univariate COX regression analysis, aging, abnormal troponin I levels, mean arterial pressure less than 70 mmHg, decreased SPO2, increased pulse rate, increased inflammatory factors (Procalcitonin and C-Reactive protein), and the occurrence of dysrhythmia at the time of admission had a significant association with increased mortality risk among COVID-19 patients in the hospital (*p*<0.05) (Table 2).

**Table 2:** Factors related to in-hospital mortality based on univariate and multivariable Cox Proportional Hazards Regression Model

**Variables**	**Crude HR** ^a^ **, 95% CI**	**P_value**	**Adjusted HR, 95% CI**	**P-value**
Age (yrs)	1.03 (1.02–1.04)	<0.001	1.02 (1.01–1.04)	<0.001
Sex (Men)	1.14 (0.85–1.53)	0.370	0.94 (0.61–1.47)	0.815
Troponin I (ng/mL)				
≤0.03	Reference	-	Reference	-
>0.03	2.73 (2.04–3.66)	<0.001	1.67 (1.08–2.56)	0.019
MAP ^b^ (mmHg)				
70-100	Reference	-	Reference	
<70	3.45 (2.01–5.86)	<0.001	0.68 (0.30–1.54)	0.358
>100	0.64 (0.30–1.38)	0.260	0.41 (0.14–1.14)	0.090
Mean of PR ^c^	1.03 (1.01-1.04)	<0.001	1.02 (1.01–1.04)	<0.001
Mean of SPO2 ^d^	0.92 (0.90–0.93)	<0.001	0.91 (0.88–0.94)	<0.001
Dysrhythmia in admission (YES)	1.79 (1.28–2.50)	0.001	1.72 (1.11–2.65)	0.014
Hemoglobin (g/dL)	0.93 (0.87–1.005)	0.072	0.93 (0.86–1.01)	0.131
C-Reactive Protein (mg/L)	1.003 (1.001–1.006)	0.003	1.002 (0.99–1.004)	0.138
Procalcitonin (ng/L)	1.02 (1.01–1.04)	<0.001	1.02 (1.008–1.04)	0.003

^a^Hazard Ratio; ^b ^Mean Arterial Pressure; ^c^Pulse Rate; ^d^Oxygen saturation measured by pulse oximetry; The model was fitted based on Schoenfeld residual test for the evaluation of proportional hazards assumption with P-value=0.179.

Based on the results of the adjusted cox regression model and considering the effect of other variable, we observed that increased age (HR=1.02, 95% CI=1.01-1.04, *p*<0.001), increased mean pulse rate (HR=1.02, 95% CI=1.01-1.04, *p*<0.001) and increased procalcitonin as an inflammatory factor (HR=1.02, 95% CI=1.008-1.04, *p*=0.003) were associated with increased risk of in-hospital mortality. However, a rise in SPO2 was asociated to reduced risk of in-hospital mortality among patients (HR=0.91; 95% CI: 0.88-0.94; *p*<0.001). Also, it was found that abnormal troponin I level in hospitalized COVID-19 patients increased the risk of in-hospital mortality by 67% (HR=1.67, 95% CI=1.08-2.56, *p*=0.019). Based on the results, dysrhythmia on admission among COVID-19 patients increased the risk of in-hospital mortality by 72% (HR=1.72, 95% CI=1.11-2.65, *p=*0.014) compared to other COVID-19 patients without dysrhythmia (Table 2). 

## Discussion

The current study is one of the few studies that investigate abnormal troponin I levels in COVID-19 patients to this extent and its further connection states to the outcome of the disease. According to the results acquired by this study, abnormal troponin I level is an independent prognostic factor to determine the mortality rate or survival length of COVID-19 patients. Subsequently, the mortality rate of patients with abnormal levels of troponin I is 67% higher than other patients. Many conditions, for instance, Acute coronary syndrome, Myocarditis, Pericarditis, Tachycardia, Heart failure, Shock, Pulmonary embolism, Pulmonary hypertension, Renal failure, Cerebrovascular Accident, and Sepsis can lead to a rise in troponin I levels [18]. Moreover, any of the conditions mentioned above can happen to a COVID-19 patient. The precise mechanism of the troponin levels rise among sepsis cases remains unclear; however, its roots can be traced to cytokine storms as well as rigorous immune response, which exist in critically ill COVID-19 patients to some extent [19]. Serum troponin I levels can also be of great importance in determining the manifestation of cardiovascular conditions. In addition, the importance of serum troponin I level’s role as a mortality predictor amongst COVID-19 patients has been uttered in several studies [20]. Moreover, COVID-19 can affect the cardiovascular system even in the absence of any symptoms of pulmonary involvement [21]. Therefore, considering the possibility of cardiovascular complications and other causes of increased troponin levels in COVID-19 patients, the need to evaluate the prognostic value of troponin I has recently been discussed.

In addition, the findings of this study are consistent with other studies performed on this topic, all of which confirm the prognostic value of examining troponin I levels in determining the prognosis of these patients [22, 23]. Traditionally, troponin I has been utilized as one of the most important biomarkers in the detection of cardiac impairment, particularly in the cases of inflammation of the heart muscle (myocarditis), acute cardiac injury, and coronary artery disease. Furthermore, according to recent studies, one of the most common cardiovascular complications in COVID-19 patients has been Acute Cardiac Injury [24]. Also, COVID-19 can increase the probability of thrombosis by causing dysrhythmias and changes in coagulation factors and when combined with cardiac involvement it will lead to a rise in troponin I levels, and consequently, increased mortality, and decreased in-hospital survival rates [25, 26]. Another interesting finding obtained in this study, which had not been elaborated in as much detail in the past, is a 2% increase in mortality per unit rise in mean pulse rate (Pulse/min). Previous studies have also shown that increased pulse rate is an independent prognostic factor to predict cardiovascular, non-cardiovascular, and all-cause mortality [27]. Both Dysrhythmia and Fatal tachycardia are common features of COVID-19, both secondary to fever and sepsis and in the context of heart tissue involvement and ischemia [25]. Therefore, the presence of tachycardia in COVID-19 patients seems to act as a simple and useful indicator in predicting the disease prognosis. Furthermore, it might be possible to use electrocardiographic findings proven to be significantly associated with patient mortality in the prior studies [28] along with troponin I levels to achieve an improved prediction of disease outcome.

Procalcitonin is a precursor of calcitonin hormone, which is secreted by Para-follicular thyroid cells and its secretion primarily rises in the course of bacterial infections [29]. It was previously thought that procalcitonin would indicate the presence of viral co-infections [30]. However, some recent studies have also attributed procalcitonin’s increase to secondary to severe viral pneumonia and sepsis [31, 32]. There are also some other studies that have reported elevated procalcitonin levels in some patients as an indicator of their greater chances of developing a severe form of COVID-19 by five times when compared to other patients [33]. Procalcitonin has also been introduced as an important indicator, which has high sensitivity and specificity in diagnosis and determining the prognosis of acute heart failure [34]. Taking all the above factors into account regarding the value of this time finding aspect along with troponin and bacterial co-infection consideration, it becomes more obvious that we can look at this cardiac biomarker as a predictor of acute heart failure in the course of COVID-19. Consequently, all the aforementioned points should be considered to reduce patient mortality through timely intervention.

In a study conducted by Shenoy *et al*., [35] it has been indicated that cellular hypoxia assists further infiltration of SARS-CoV-2 virus into human cells via upregulating the expression of ACE2 receptor and therefore, exacerbates fibrosis and other detrimental effects caused by the virus in the lung tissue. Also, considering the drop in the level of blood oxygen as a common complication in COVID-19 patients, this study demonstrates that an increase in the blood oxygen saturation level by itself can be used as an effective tool against COVID-19-related mortality. Therefore, for the purpose of better prognosis of patients, blood oxygen levels should remain at the expected range as much as possible. 

The results obtained from the study showed that abnormal levels of troponin I correlate with in-hospital mortality and can be used as a suitable indicator to predict the outcome of COVID-19 patients. Due to the high prevalence of cardiac complications in these patients, it is highly suggested to monitor and control the cardiac biomarkers along with other clinical factors upon patient’s arrival at the hospital.

This study, similar to many other retrospective studies, has certain limitations. The most important limitation to mention is that the criterion for laboratory studies done in this study is a blood sample received in the initial 24 hours after patient admission to the hospital and serial blood samples were not ordered for all patients during hospitalization to measure troponin levels. Therefore, patients who subsequently developed cardiac tissue damage as a result of enhanced troponin levels, as well as other causes, were not considered. Furthermore, due to limited hospital facilities amid the current pandemic circumstances, it was not possible to perform further cardiac assessments such as echocardiography for all patients. Hence, possible causes of increased troponin levels in patients have not been evaluated. In addition, the study population solely included hospitalized patients who had all suffered from Moderate to Severe forms of the disease, and therefore Mild to Moderate or Asymptomatic cases have not been incorporated in the study of the disease. Additionally, this study followed the patients from the time of hospitalization until the time of discharge or death in the hospital. 

## Declarations

### Ethical approval and consent to participate:

Ethical approval has been granted by Deputy for Research Affairs, Shahid-Beheshti University of Medical Sciences (IR.SBMU.RETECH.REC.1399.684).

### Consent for publication:

The datasets of the current study are not publicly available but are available via the corresponding author upon reasonable request.

### Conflict of interest:

The authors declare that they have no conflict of interests.

### Funding:

This study was supported by a grant from the Prevention of Cardiovascular Disease Research Center with a grant number of 25886.

### Authors Contribution:

The conception or design of the work was done by MHA, MS. Data was collected by FNA and AT, and analyzed by NT. Results were interpreted by MHA, MS, RM, MPM, FO, AP and RS. Drafting the article was done by AT and MS and revised by MHA and all authors read and approved the manuscript.

### Acknowledgment:

The authors would like to thank the ICU and CCU medical and nursing personnel of Imam Hossein Hospital, Shahid Beheshti University of Medical Sciences; Dr.Ainaz Samadi, Dr.Amir Heydari , Dr.Mahboubeh Ghazanfarabadi and Dr.Moazzameh Aghamohammadi, Effat Taheri, Faezeh Nesaei, Faezeh Fakour, Ghazaleh Aman-Abadi, Ghodsi Najjari, Golnoush Mortezaei, Maedeh Sayyad and Vida Torabi who participated in this project and helped us in performing this project.
